# Google-Earth Based Visualizations for Environmental Flows and Pollutant Dispersion in Urban Areas

**DOI:** 10.3390/ijerph14030247

**Published:** 2017-03-02

**Authors:** Daoming Liu, Sasa Kenjeres

**Affiliations:** 1 Safety and Emergency Laboratory, Shanghai Advanced Research Institute, Chinese Academy of Sciences, No.99 Haike Road, Pudong New District, Shanghai 201210, China; 2 University of Chinese Academy of Sciences, Beijing 100049, China; 3 Transport Phenomena Section, Department of Chemical Engineering, Faculty of Applied Sciences and J. M. Burgerscentrum for Fluid Mechanics, Delft University of Technology, Van der Maasweg 9, 2629 HZ Delft, The Netherlands

**Keywords:** computational fluid dynamics, visualization, Google Earth, environmental pollution, KML

## Abstract

In the present study, we address the development and application of an efficient tool for conversion of results obtained by an integrated computational fluid dynamics (CFD) and computational reaction dynamics (CRD) approach and their visualization in the Google Earth. We focus on results typical for environmental fluid mechanics studies at a city scale that include characteristic wind flow patterns and dispersion of reactive scalars. This is achieved by developing a code based on the Java language, which converts the typical four-dimensional structure (spatial and temporal dependency) of data results in the Keyhole Markup Language (KML) format. The visualization techniques most often used are revisited and implemented into the conversion tool. The potential of the tool is demonstrated in a case study of smog formation due to an intense traffic emission in Rotterdam (The Netherlands). It is shown that the Google Earth can provide a computationally efficient and user-friendly means of data representation. This feature can be very useful for visualization of pollution at street levels, which is of great importance for the city residents. Various meteorological and traffic emissions can be easily visualized and analyzed, providing a powerful, user-friendly tool for traffic regulations and urban climate adaptations.

## 1. Introduction

The continuous degradation of urban air quality is closely related to intensive worldwide urbanization. It is estimated that in coming decades almost 70% of the world’s population will live in urban areas [1]. A rapid pace of urbanization is associated with an increase of pollutant concentrations, originating from industrial and traffic emissions. This increase in source emissions in combination with extreme climate or weather events requires the development and application of state-of-the-art numerical simulation tools, which will be able to better understand local environmental conditions to support the development of sustainable environmental solutions for urban areas [2,3]. Sustainable solutions for urban areas usually include a minimization of air and water pollution, as well as a reduction of energy and water use. To achieve the required reductions in air and water pollution, various scenarios including local meteorological, climate, geographical and emission conditions need to be taken into account in numerical simulations. In the present study, to provide detailed information of air pollution in urban areas generated by traffic emissions, we apply our recently developed computer simulation code that merges computational environmental fluid mechanics with computational atmospheric chemistry. Both parts of this code are based on solving a discretized system of full transport equations for conservation of mass, momentum and reactive species (pollutants). This computational framework represents an approach, which integrates computational fluid dynamics and computational reaction dynamics. Discretization of transport equations is based on a finite-volume method for general non-orthogonal geometries. One of the crucial aspects of the development of such advanced numerical simulation tools is their detailed testing and validation against available experimental studies such as [4,5,6,7] wherein we demonstrated the capabilities of the modeling approach presented here. Note that resolving a simulation in such detail (including buildings, vegetation, and terrain) as an integral part of the Computational Fluid Dynamics/Computational Reaction Dynamics approach is significantly more accurate in predicting local flow patterns, turbulence intensity and distributions of the passive or reactive scalars than other simplified methods discussed in the literature. The simplified models include the DEGADIS and SLAB models for dense gas dispersion [8], the BM model [9], AERMOD and CALPUFF models supported by the US’ EPA [10], ADMS model developed by Cambridge Environment Research Consultants [11], etc. The majority of these models are based on a relatively simple standard or slightly extended Gaussian approach for predictions of pollutant dispersion. 

In addition to accurate predictions, it is also crucial to have advanced visualization tools for interpretation and extraction of detailed information from the simulation results obtained. In the past, we have used various stand-alone advanced visualization tools such as ParaView, Tecplot, Advanced Visual System, Visit, etc. Despite numerous features that are commonly used to show and analyze CFD datasets, in all these applications we are missing a photo-realistic representation of simulated cases. Furthermore, to be able to use these above-mentioned stand-alone visualization tools, users need to have rather advanced technical or engineering skills. In the present study, we propose to circumvent some of these limitations by developing a novel visualization method that is based on the Google Earth. Our goal is to have a relatively simple, cost- and user-friendly visualization tool, which will make possible to generate a photo-realistic environment for analysis of generic CFD/CRD datasets.

Google Earth, provided by Google Inc. (Mountain View, CA, USA), is free of charge and is the most popular virtual globe software. In addition to the standard use as a geo-browser for exploring spatially referenced data, it can also be applied to serve as a visualization platform for various scientific disciplines. In recent years, some scientific types of visualizations based on Google Earth have been reported, including leaking gas diffusion [12,13], meteorological satellite data [14], seismic tomographic data [15], meteorological data [16], structure of the earth’s lithosphere [17], remote sensing data [18], hurricane disasters [19] and land use land cover dataset [20]. 

Some of the core elements of Google Earth/Sketchup/Maps based visualizations that are similar to the present study were shown in [12,13,16]. In [12], a relatively simple Gaussian dispersion models (i.e., “plume” and “puff” variants) were used to predict spreading of the unexpected gas (carbon monoxide) leak accident. The results were visualized as the 2D projections of the pollution regions in Google Maps. In [13] a different approach was followed—the Google Sketchup was used to extract the coordinates of buildings, which are then used for CFD simulations (based on a commercial Fluent software) of a gas leakage (methane) within the Sun Yat-sen University East campus. The entire post-processing was performed within the Fluent. Finally, in [16], authors used Google Maps/Earth to visualize the data originating from meteorological models. The primary focus was on the wind (generated by 3DWF weather forecasting model) visualization in complex terrains, but without providing any details behind algorithms for conversion of data and their visualization. 

The present work extends previously mentioned studies in the sense that it provides ways to convert and to visualize results of integrated CFD/CRD approach (which include velocity fields, pressure, turbulence parameters as well as the reactive scalar distributions) used to predict levels of the local pollution within complex urban areas in the Google Earth. Moreover, we also report on the most important steps in constructing the integrated algorithm as well as on details in generating various geometrical elements used for visualization of the data. This is especially important for the code/algorithm developers who want to adopt the presented approach for their needs.

Finally, capabilities of developed tools will be demonstrated in a case of photochemical smog formation for a selected neighborhood in the city of Rotterdam (The Netherlands).

## 2. Materials and Methods

In this section, we present systematically the overall framework and key algorithms of the developed visualization tool based on Google Earth for showing results obtained by CFD simulations. It contains the majority of the features used by standalone CFD visualization packages, which are now directly implemented in our in-house code based on the Java language.

### 2.1. Overall Framework

**Figure 1:**
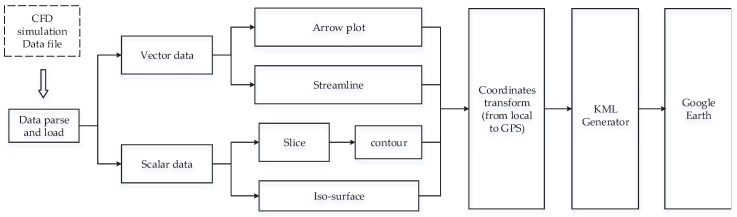
The flow chart of the developed algorithm with the most important visualization techniques used for showing scalar and vector data formats, which are then converted to the GPS coordinate system, and through generation of the Keyhole Markup Language KML objects, finally, displayed in the Google Earth.

The overall framework of the data conversion and visualization approach is shown in Figure 1. The entire process can be divided into three major steps: (I) parsing and loading of the data generated by CFD simulations; (II) selection of the targeted visualization methods (vectors, contours, streamlines, isosurfaces, etc.) and data processing with the appropriate algorithm for their generation; (III) conversion of all coordinates defined in the local simulation domain to the coordinates based on the global positioning system (GPS) and a final generation of the KML files.

### 2.2. KML File Format

The KML is a file format used to display geographic data in Google Earth and also an international standard maintained by the OGC (Open Geospatial Consortium) since 2008. It allows the user-defined data to be imported and graphically edited in Google Earth directly. A KML file is constructed with many KML elements (the entire class tree of KML elements can be found in [21]). Among them, the most-often used are “Geometries” including “Point”, “LineString”, “Polygon”, “LinearRing”, “MultiGeometry”, “ColorStyle” including “IconStyle”, “LineStyle” and “PolyStyle”, and “Placemark”. When making a tour or movie, “Camera”, “TimeSpan” and “TourPrimitive” will be used. 

### 2.3. Key Algorithms

#### 2.3.1. Data Generation: An Integrated CFD/CRD Approach

The details of the fully integrated CFD/CRD approach, with all equations, are given in [22]. Here we will give just a concise overview of the full transport partial differential equations based on conservation of the mass, momentum, energy and concentration of species. To have a fully closed system of equations, due to the turbulent nature of the considered flow, the Reynolds-Averaged-Navier-Stokes (RANS) turbulence closure is used, which is based on the two equations eddy-viscosity modeling approach. Similarly, the chemical reactions describing the photochemical smog generation are completed as a generic-reaction-set (GRS) model. The general form of all transport equations, which is based on the finite-volume discretization approach, is of the convection-diffusion-source-sink type and can be finally written in index notation as:

(1) $$\int_{V}\frac{\partial \Phi }{\partial t}dV=\int_{S}\left(\Gamma_{\Phi }\frac{\partial \Phi }{\partial x_{j}}-\Phi U_{j}-\bar{\phi u_{j}}\right)n_{j}dS\pm \int_{V}S_{\Phi }dV$$

Here, the “Φ” is an arbitrary transport variable (e.g., the averaged velocity (*U_j_*) or concentration of species (*C*^(*n*)^), with *n* = NO, NO_2_, O_3_, etc.); *V* is the volume of the numerical mesh segment; *S* is the surface of the cell-face of the numerical mesh segment; *n_j_* is unit vector perpendicular to the cell-face of the mesh segment; ($\bar{\phi u_{j}}$) is the correlation representing interactions between fluctuating components (turbulence contributions). In the standard RANS approach, the first term on the left-hand side (LHS) of the equation is zero. The terms on the right-hand side (RHS) represent the molecular diffusion, convection, turbulent diffusion and source/sink terms, respectively. The diffusive terms are discretized by the second-order central-differencing scheme (CDS), whereas the convective terms are represented as second-order linear (LUDS) or quadratic upwind differencing (QUDS) schemes. The coupling between the velocity and pressure is calculated iteratively with the Semi-Implicit Method for Pressure Linked Equations (SIMPLE). 

#### 2.3.2. Data Load

The dataset of CFD flow simulation is usually stored in the formats supported by the standalone general-purpose visualization software, e.g., Tecplot, ParaView, Visit, AVS, etc. A majority of supported formats include the spatial coordinates of the control volume nodes and values of vector and scalar components at the control volume centers. This information is sufficient to have a full 3D description of the all dependent variables (e.g., velocity field, concentrations, etc.). The first step is to parse the data file and load the CFD data. Due to the usually large size of the data file, we adopt an interactive design of the interface code. In this way, we need to parse and to load the big data file only once. This design makes the entire process computationally quite efficient (e.g., about 1 min is required to load 1.6 GB of data written in the ASCII Tecplot data format on a standard laptop system (Win7, Intel(R) Core(TM) i7-4710MQ, 16 GB RAM). After this initial loading of the data, because of the efficiency of the visualization algorithms, various visualization techniques can be interactively performed, resulting in very fast performances (not longer than 1–2 s). The results of a simulation are usually divided into two groups: the vector data (velocity, turbulent heat flux, turbulent concentration flux, etc.) and the scalar data (pressure, temperature, concentration, etc.).

#### 2.3.3. Vector Data Visualization Algorithms

**Figure 2:**
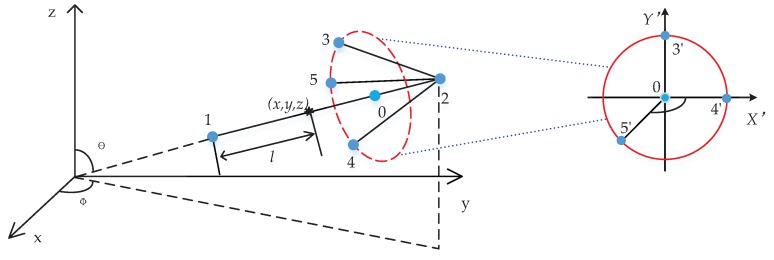
The sketch illustrating the major steps in constructing three-dimensional arrows for representation of the velocity vectors.

For the representation of the vector data, one of the most commonly used visualization methods is to plot an arrow at a given location with a particular length (and color) and direction. Despite its simple definition, plotting vectors in 3D still require some additional information. In the present work, for vector plotting, we adopt the convention that the vector heading has the form of a pyramid. A sketch showing this approach is depicted in Figure 2. Points (1) and (2) indicate the beginning and end of the vector baseline. The central location is associated with the (*x*, *y*, *z*) coordinates of the centre of the computational control volume, whereas points (1) and (2) are obtained by calculating the vector magnitude and applying the proper scaling. Then we define a location of the point (0)—again based on the proper scaling and a new parameter (*f*) that indicates a ratio between the length of the vector heading and its total length. Next, a virtual circle with its centre at the point (0) and with a radius (*r*), perpendicular to the vector baseline, is constructed. Finally, three points (3, 4, 5) are specified at the circle. The details of calculating required coordinates of these points are provided next.

(2) $$x_{1}=x-l\cdot sin(\theta )\cdot cos(\phi ),y_{1}=y-l\cdot sin(\theta )\cdot sin(\phi ),z_{1}=z-l\cdot cos(\theta )$$

(3) $$x_{2}=x+l\cdot sin(\theta )\cdot cos(\phi ),y_{2}=y+l\cdot sin(\theta )\cdot sin(\phi ),z_{2}=z+l\cdot cos(\theta )$$

(4) $$x_{0}=x+f\cdot l\cdot sin(\theta )\cdot cos(\phi ),y_{0}=y+f\cdot l\cdot sin(\theta )\cdot sin(\phi ),z_{0}=z+f\cdot l\cdot cos(\theta )$$

Coordinates of the points (3’, 4’, 5’) can be easily obtained, i.e., (r,0,0),(0,r,0) and $\left(-\frac{r}{\sqrt{2}},-\frac{r}{\sqrt{2}},0\right)$ respectively. To calculate the coordinates of points (3, 4, 5), a coordinates system transformation needs to be applied as follows:

(5) $$\begin{array}{c} \left[\begin{array}{ccc} x_{i} & y_{i} & z_{i} \end{array}\right]=\left[\begin{array}{ccc} x_{i}^{\prime } & y_{i}^{\prime } & z_{i}^{\prime } \end{array}\right]R^{-1}T^{-1},T=\left[\begin{array}{cccc} 1 & 0 & 0 & 0\\ 0 & 1 & 0 & 0\\ 0 & 0 & 1 & 0\\ x & y & z & 1 \end{array}\right]\\ R_{x}=\left[\begin{array}{cccc} 1 & 0 & 0 & 0\\ 0 & \frac{c}{\sqrt{b^{2}+c^{2}}} & \frac{b}{\sqrt{b^{2}+c^{2}}} & 0\\ 0 & \frac{-b}{\sqrt{b^{2}+c^{2}}} & \frac{c}{\sqrt{b^{2}+c^{2}}} & 0\\ 0 & 0 & 0 & 1 \end{array}\right],R_{y}=\left[\begin{array}{cccc} \frac{\sqrt{b^{2}+c^{2}}}{\sqrt{a^{2}+b^{2}+c^{2}}} & 0 & \frac{a}{\sqrt{a^{2}+b^{2}+c^{2}}} & 0\\ 0 & 1 & 0 & 0\\ \frac{-a}{\sqrt{a^{2}+b^{2}+c^{2}}} & 0 & \frac{\sqrt{b^{2}+c^{2}}}{\sqrt{a^{2}+b^{2}+c^{2}}} & 0\\ 0 & 0 & 0 & 1 \end{array}\right],R=R_{x}R_{y} \end{array}$$

where (*x*, *y*, *z*) denotes the coordinates of the centers of control volumes; (*x*_1_, *y*_1_, *z*_1_) and (*x*_2_, *y*_2_, *z*_2_) denote the coordinates of the two end points of the arrow respectively; (*x_i_*, *y_i_*, *z_i_*) denotes the coordinates of points 3, 4, 5; (*x_i_’*, *y_i_’*, *z_i_’*) denotes the coordinates of points 3’, 4’, 5’; (*x*_0_, *y*_0_, *z*_0_) denotes the coordinates of the cross-point between the arrow and the circle plane; *r* denotes the radius of the virtual circle; *l* denotes half of the length of the arrow; *f* is a constant factor that is used to locate the circle and it can decide the angle of the arrow head with *r*; θ and φ denote the wind direction at the control volume centre. *T* denotes the translation matrix, *R* denotes the rotation matrix, *R_x_* and *R_y_* denote rotation matrices around the *x*-axis and *y*-axis respectively, and (*a*, *b*, *c*) indicates the velocity at the centre of the control volume. Now, the vector head can be directly converted into a tetrahedron employing the intrinsic KML function “polygon”. Displaying the vectors for all control volumes will result in a large size KML file that Google Earth will not be able to show smoothly. The solution is to pre-select some characteristic planes of interest and then to display vectors at these locations. In addition to the vectors, streamlines and streamlets are often used to visualize the velocity field. Here we briefly discuss their implementation in the present algorithm. In the first step, the mass-less particles are released at initial locations, and the closest control volumes are identified. Then, a value of the velocity component at these locations is calculated by applying tree-linear interpolation methods for the control volume vortices. In the second step, new locations of the mass-less particles (tracers) are re-calculated by multiplying the local velocity with a characteristic time step (here we apply the 2nd order Runge-Kutta method):

(6) $$x_{e}=x_{s}+\Delta t\cdot v_{x},\;y_{e}=y_{s}+\Delta t\cdot v_{y},\;z_{e}=z_{s}+\Delta t\cdot v_{z}$$

where (*x_s_*, *y_s_*, *z_s_*) denotes the start point, (*x_e_*, *y_e_*, *z_e_*) indicates the end point, Δt denotes the time step, and (*v_sx_*, *v_sy_*, *v_sz_*) indicates the velocity of the start point *S*. Then find the closest control volume for the end point E and calculate the velocity (*v_ex_*, *v_ey_*, *v_ez_*) by tri-linear interpolation. To complete this second step, calculate the end of the coordinates of point E again with the averaged velocity, as follows:

(7) $$x_{e}=x_{s}+\Delta t\cdot \left(v_{sx}+v_{ex}\right)/2,\;y_{e}=y_{s}+\Delta t\cdot \left(v_{sy}+v_{ey}\right)/2,\;z_{e}=z_{s}+\Delta t\cdot \left(v_{sz}+v_{ez}\right)/2$$

In the third step, steps one and two are repeated by considering the latest positions till the pre-specified number of time steps or the boundary of the domain are reached. In the fourth final step, the KML file is generated containing the “String Lines”. Here, each string line includes the entire history of tracer locations (from the initial seeding point till the latest position). The stream-ribbon and stream-tube can also be generated based on the streamline and the same idea used in the arrow plot above for finding the particular points. The stream-ribbon needs just one more particular point for each time in the streamline and the stream-tube generally needs at least six points for a smoothing effect. In addition to the streamlines, the streamlets can also be shown. The streamlets can be interpreted as short streamlines segments with the length proportional to the velocity magnitude and for which uniformly distributed seeding particles are used. 

#### 2.3.4. Scalar Data Visualization Algorithms

**Figure 3:**
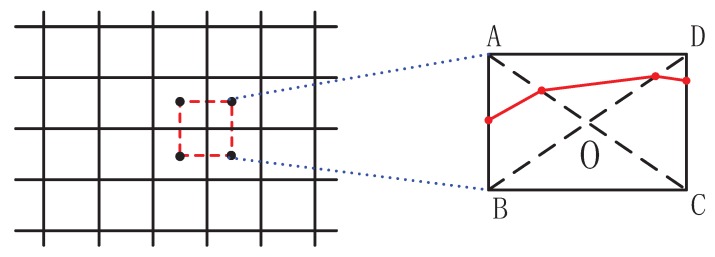
The underlying mesh that connects the centers of the control volumes (**left**) and a zoom-in (**right**) showing interpolation within the control volume (defined with vortices A-B-C-D) used to construct the characteristic isolines (red lines).

For visualization of the scalar data, here we select two of the most popular methods: characteristic contours in pre-selected planes (2D data) or a full isosurface or wire-frame representation (3D data). The contours are obtained in the following way. Here, each rectangular mesh that connects centers of the control volumes is divided into four triangles (see Figure 3). The procedure of obtaining these locations is provided next. Firstly, calculate the point (1) on the edge AB (according to the following equation) by a linear interpolating method with the values of end points (*A*, *B*), then find the point (2) on edge AO by the same method, then connect 1 and 2 into a line, and finally, merge all the lines in all other triangles:

(8) $$C_{i1}=\frac{S_{t}-S_{B}}{S_{A}-S_{B}}C_{iA}+\frac{S_{A}-S_{t}}{S_{A}-S_{B}}C_{iB}$$

where *C_i_*_1_ denotes the coordinates of point (1) and *i* indicates possible *x*, *y* or *z*. *C_iA_* and *C_iB_* denote the corresponding coordinates of *A* and *B* respectively. *S_t_* denotes the scalar threshold value, *S_A_* and *S_B_* indicate the scalar value of *A* and *B*, respectively.

**Figure 4:**
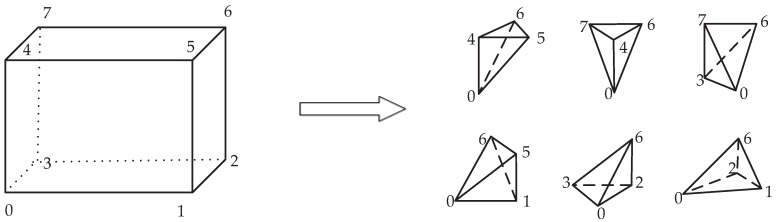
Division of a cuboidal control volume into 6 tetrahedrons required in modified marching tetrahedron approach used for plotting of the isosurfaces or isoframes of scalar variables.

For a three-dimensional representation, the isoframes or isosurfaces of the scalar variables are often used. The method presented above for finding the contour plots is extended to the third spatial coordinate by applying the modified marching tetrahedron approach [23]. The advantage of this method is that we can use a small lookup table. This is achieved by dividing the cuboidal control volume into 6 tetrahedrons as illustrated in Figure 4. 

Contours are calculated for each of the individual four faces of the tetrahedron using the contour algorithm described above, and all contour lines are merged to form a polygon. Then, this operation is repeated for all remaining control volumes and their tetrahedral subsets. In the last step, including all polygons with elements “Polygons” and “Linear Ring”, the KML file is generated.

#### 2.3.5. Transformation of Coordinates

The last step is to perform coordinate transformation between the local arbitrarily selected coordinate system, used in CFD simulations, to the World Geodetic System (WGS84) utilized by the Global Positioning System (GPS) within Google Earth. Here, we address the conversion for results of simulations of environmental flows and turbulent dispersion of pollutants at scales of a few kilometers (city district/neighborhood scales). With the assumption that earth is a simple sphere, the resulting coordinate transformations are performed:

(9) $$lat=lat_{0}+arcsin\left(\frac{Y-y_{0}}{R}\right)\frac{360}{\pi },lon=lon_{0}+arcsin\left[\frac{X-x_{0}}{R\cdot cos\left(lat_{0}\right)\frac{\pi }{360}}\right]\frac{360}{\pi },alt=alt_{0}+Z-z_{0}$$

where, (*X*, *Y*, *Z*) denotes the CFD ENU coordinates (local East, North and Up); (*lat*, *lon*, *alt*) denotes the GPS coordinates; (*x*_0_, *y*_0_, *z*_0_) are the ENU coordinates of one given point in the computational domain; (*lat*_0_, *lon*_0_, *alt*_0_) are the GPS coordinates of the given point; *R* denotes the averaged radius of the earth.

However, if the simulated area is larger than ~20 km this assumption can introduce some errors. In such cases, the accuracy of the coordinate transformation can be improved by treating the earth as an ellipsoid. In such a situation, a method proposed in our previous study can be applied [12]. In the CFD simulations, the down-wind direction is usually annotated as the positive *x*-coordinate direction. Before using the equations given above, we apply a rotation of the coordinate system from the local wind direction reference system to the local ENU-type coordinate system by the following equations:

(10) X=x⋅cos(n)+y⋅sin(n),Y=y⋅cos(n)−x⋅sin(n),Z=z

where (*X*, *Y*, *Z*) denotes ENU coordinates; (*x*, *y*, *z*) denotes local coordinates with *x* as the wind direction; *n* is the rotation angle.

## 3. Results and Discussion

**Figure 5:**
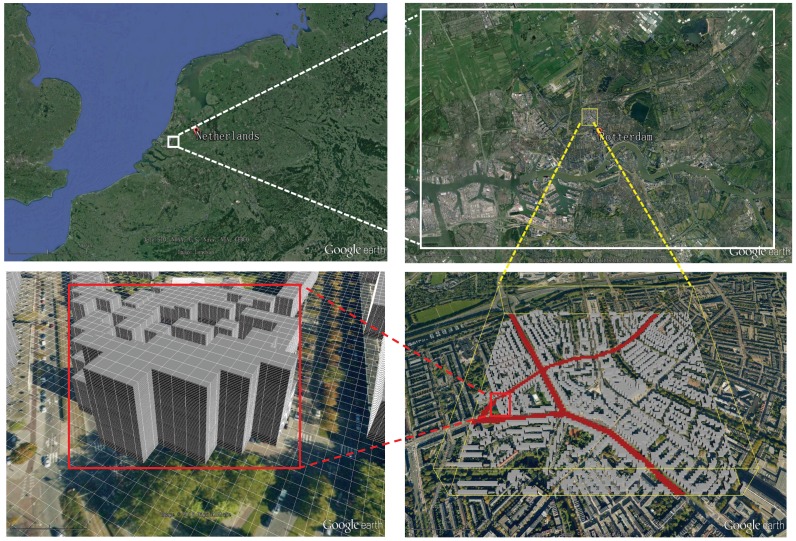
The set-up of the simulated case shown in the Google Earth: region (The Netherlands) scale; city (Rotterdam) scale and neighborhood (the “Oude Noorden”) scale including computational mesh and obstacles representing parts of buildings. In total, ~1800 obstacles are included in simulations. The traffic emission sources (including the three major roads) are marked in red. The region within the yellow box is geometrically reconstructed and simulated in details.

In this section, we demonstrate an application of the above-described algorithms to show results of an integrated CFD and CRD approach in simulating flow, turbulence, and dispersion of reactive pollutants in a real scale urban area, and how generated results are finally visualized in the Google Earth. Here we present data calculated in [22] for traffic pollution and ozone distribution in one neighborhood of the city of Rotterdam (The Netherlands, Figure 5). The region of interest covers a zone of 1 × 1 km located in the “Oude Noorden” neighborhood with three main traffic arteries: Bergselaan, Schieweg and Bergweg streets (which are shown in red color—where the source emissions of NO and NO_2_ are defined). The GPS coordinates of the left bottom corner of the simulated domain are (51.928640, 4.461468, 0) (in decimal degrees), where the “0” indicates the ground level in the *z*-direction. The buildings of selected neighborhood are generated from the digital elevation map (DEM) dataset obtained from the city officials. 

**Figure 6:**
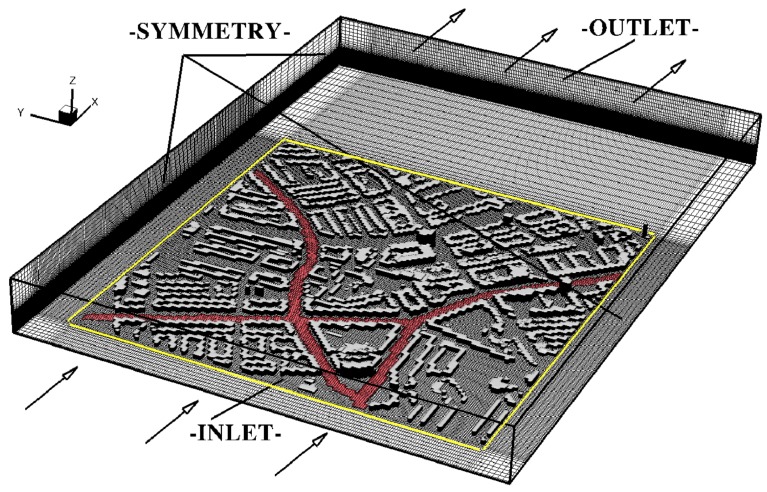
The simulated Computational Fluid Dynamics domain with imposed boundary conditions shown in the stand-alone graphical environment (Tecplot). The INLET boundary conditions are imposed to mimic selected meteorological conditions that include the wind intensity, its direction, and intensity of wind fluctuations. The OUTLET boundary assumes a zero gradient condition (in the wind direction) for all variables. The SYMMETRY boundaries impose that there is not flow perpendicular to the boundary. The red segments indicate the main traffic emission sources (roads). The numerical mesh used is also shown in characteristic planes (in total, 5.4 × 10^6^ control volumes are used for computations). The yellow box indicates the extracted sub-domain exported for Google Earth visualizations.

The environmental chemistry is simulated by applying the GRS model, which includes photovoltaic chemical reactions involving NO, NO_2_, O_3_ and Reactive Organic Compounds (ROC). The environmental fluid mechanics is simulated by applying a RANS approach to obtain velocity, pressure and turbulence variables (turbulence kinetic energy and its dissipation rate). The locally refined hexagonal numerical mesh is used with typical control volume sizes of 3.5 × 3.5 × 0.25 m in the proximity of buildings, up to 40 × 40 × 40 m for regions above the urban canopy, in the *x*-, *y*- and *z*-coordinate directions, respectively. Note that the *z*-direction indicates the vertical coordinate. Approximately 5.4 × 10^6^ control volumes are used for the entire simulation domain, and about 1800 obstacles are used to represent the buildings. The simulated CFD domain with imposed boundary conditions and numerical mesh used is depicted in Figure 6. At the inlet, approaching wind profile and intensity of the turbulence kinetic energy and its dissipation rate are defined from available meteorological measurements. The simulated scenario includes a light breeze wind conditions (~2 m/s) blowing from the West, which was based on the wind-rose map for that area for the selected time period. The symmetry boundary conditions are applied to the side and top boundaries.

**Figure 7:**
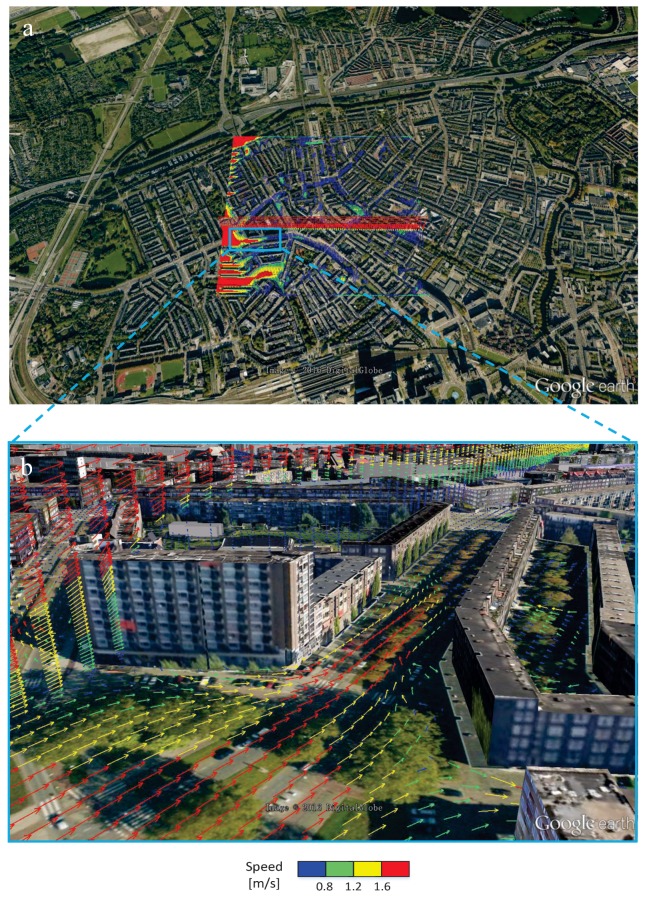
Vector representation of the wind flow in characteristic horizontal (close to the ground) and vertical planes: (**a**) overview for the entire domain at *z* = 1 m and *y* = 454 m planes; (**b**) zoom-in. The street-canyon effect (in the horizontal plane) and wakes generated behind taller buildings (in the vertical plane) can be easily observed. Note: a length of 12 m represents the reference vector of 2 m/s.

**Figure 8:**
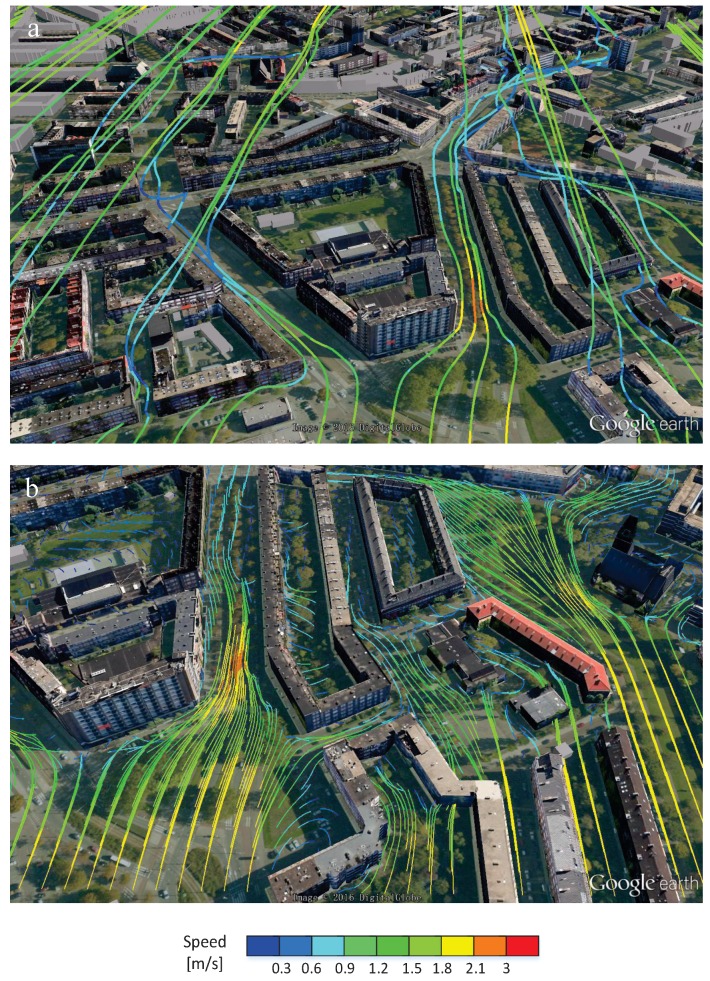
(**a**) Streamlines and (**b**) streamlets portraying the details of the wind patterns and their changes at various locations due to the presence of the buildings. Similarly to the previous figure, the red segments indicate strong wind flow regions, whereas the blue sections indicate the low wind intensity regions.

First, we demonstrate how the local wind patterns can be visualized. This information can be used to evaluate locations where the wind intensity at the pedestrian level exhibits some critical threshold values (so-called “pedestrian wind comfort”). Simulations can reveal places where the wind along the street can reach too high values. Furthermore, some mitigation scenarios can also be considered. For example, what are the effects of planted trees along the street and how the type and placing of trees can be optimized? The velocity field in the selected *z*-plane with the height of *z* = 1 m and *y*-plane where *y* = 454 m is shown in Figure 7. The top panel shows the wind field in the whole domain, whereas the bottom panel is a zoom-in showing the local wind distribution in more detail. Colors are used to denote the wind speed compared to a threshold value. The street-canyon effects (i.e., local acceleration of the wind due to blockage effects of buildings—indicated by red vectors), the local wind recirculation as well as the low wind intensity regions (indicated by blue vectors) can be easily observed. Similar information can be extracted even without showing the 3D velocity vectors. This is achieved by plotting the streamlines or streamlets, which are straightforward and intuitive ways to interpret a local wind distribution. In Figure 8, the top panel denotes streamlines with 50 seeds located in the line where *x* = 0 m and *z* = 0.75 m.

The bottom panel denotes the streamlets calculated with first seeds located in the *z*-plane where *z* = 1.8 m. For both panels, nine colors (in gradations from blue to red) are used, and they denote different velocity ranges with the threshold values 0.3, 0.6, 0.9, 1.2, 1.5, 1.8, 2.1 and 3 m/s, respectively. Now, locations with a sudden velocity increase can be easily mapped. Note that we selected just one plane for demonstration purposes, but streamlines or streamlets can be easily plotted for any arbitrary pre-selected planes.

Next, we focus on visualizations of the pollution distribution within the simulated neighborhood. Here, due to the scalar nature of variables (concentrations of chemical species), we use isolines and isosurfaces to identify their spatial distributions. The isolines are primarily useful to map concentrations of chemical species in pre-selected planes. In contrast to that, isosurfaces are used to estimate fronts of pollution spreading. With a combination of these two approaches, a detailed analysis of the local levels of pollution can be performed (e.g., to identify the most critical pollution spots).

**Figure 9:**
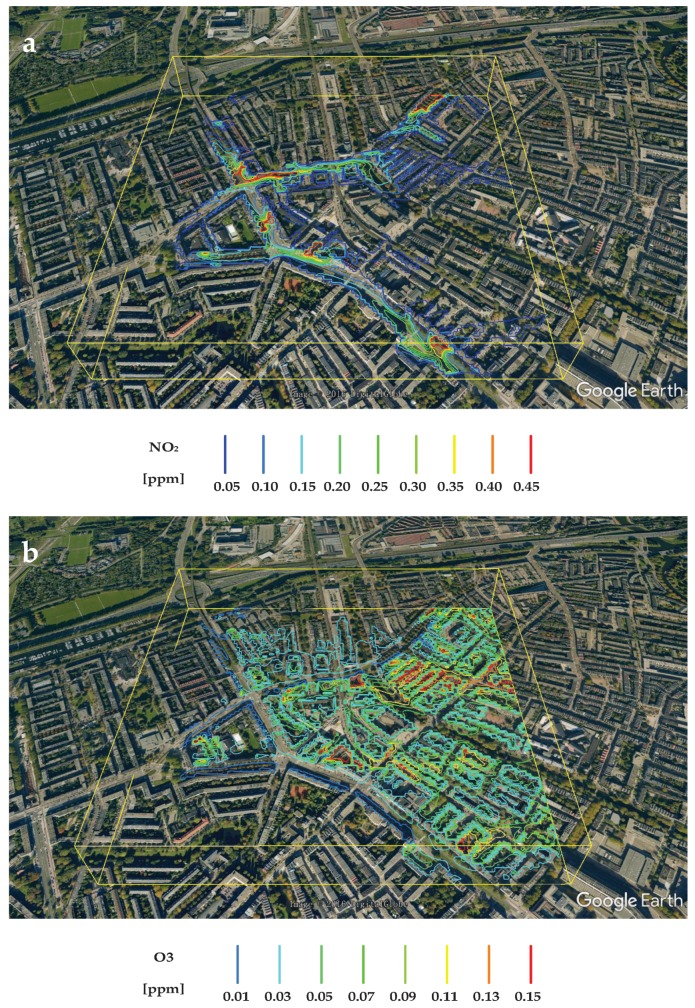
The mapping of pollution by isolines: (**a**) concentration contours of NO_2_ (indicating the locations with high emission sources, i.e., traffic) and (**b**) concentration contours of O_3_ (indicating locations with enhanced ozone distribution as result of the chemical reactions)—both in the horizontal plane at pedestrian level (*z* = 2 m).

As an example, the isolines of the NO_2_ and O_3_ are shown in *z* = 2 m plane, Figure 9. It can be seen that the maximum concentrations of the NO_2_ are observed in the proximity of the roads, which is expected since the NO_2_ is a direct product of traffic emission (Figure 9a). It is also important to see that the local concentrations of NO_2_ vary significantly along different sides of streets due to the imposed wind conditions. In contrast to the NO_2_, the ozone O_3_ levels show a significantly different behavior (Figure 9b). This is the result of the complex convection-turbulent diffusion-chemical reaction mechanisms, which are simulated within the CFD/CRD model. This additionally stresses a potential of such advanced mathematical models to map various pollution scenarios based on different meteorological (wind intensity and direction) and traffic (low, moderate or high intensity) conditions.

**Figure 10:**
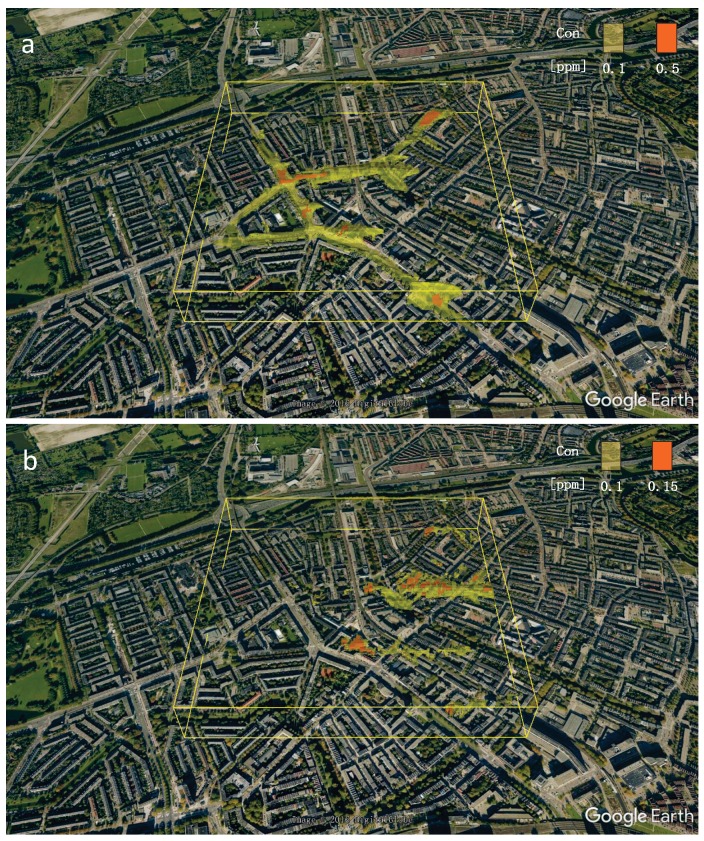
Visualization of characteristic pollution fronts by selecting some critical thresholds of species and connecting them as 3D isosurfaces of (**a**) NO_2_ and (**b**) O_3_.

**Figure 11:**
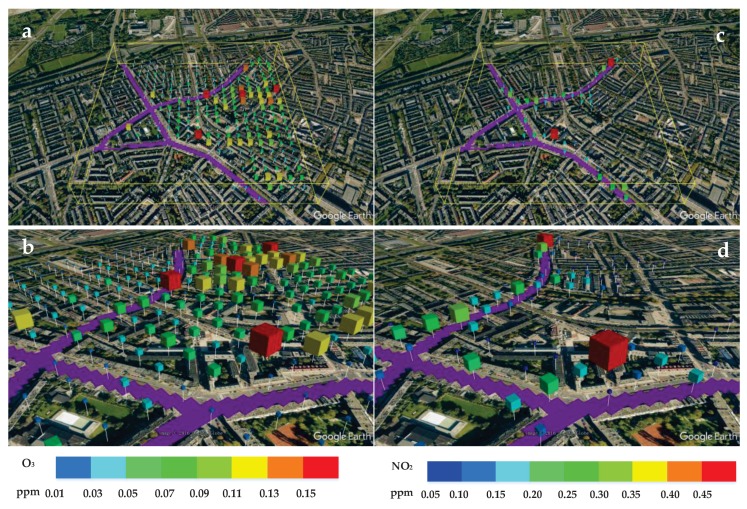
The virtual concentration sensors generated as the discrete volume objects, which size and color are proportional to the concentrations of species at given locations. Concentrations of: O_3_ (**a**,**b**); NO_2_ (**c**,**d**) across the whole domain or for particular streets, respectively.

The isosurfaces of NO_2_ (with levels of 0.5 ppm and 0.1 ppm, respectively) and O_3_ (with levels of 0.15 ppm and 0.1 ppm, respectively) are shown in Figure 10. Note that this way of presenting of results can be very useful to identify regions with increased risks for population suffering from the chronic respiratory diseases (e.g., asthma, chronic obstructive pulmonary disease (COPD), etc.) by a simple pre-specification of critical concentration thresholds for individual chemical species (e.g., NO_2_, NO, O_3_, SO_x_, particulate meter, etc.). In present work, in addition to isolines and isotherms, we also propose a simple method to visualize the local concentrations as discrete volume objects, as demonstrated in Figure 11. Here, size and color of the object define the concentration level at pre-defined locations. This method is simple for understanding and can be easily interpreted as a kind of the virtual probes/sensors at given locations. The pre-defined critical concentration thresholds can be re-scaled to provide an easy navigation through the data. For example, the red objects indicate increased and potentially harmful levels, the yellow objects indicate intermediate pollution levels, and finally, the blue objects indicate safe concentrations. This concept was used in showing the distributions of O_3_ (Figure 11a,b) and of NO_2_ (Figure 11c,d). Please note that the density of the virtual probes/monitors distribution can be easily adjusted for specific regions (e.g., along streets or in the proximity of particular buildings or crossroads, parks, hospitals, schools, etc.).

## 4. Conclusions

In the present study, we have developed an efficient tool, which enables Google Earth visualization of results obtained by CFD simulation of environmental flow and turbulent dispersion of reactive scalars in urban areas. The most common ways of representing 3D vector and scalar variables are included in this tool (3D vectors and their projections at specified planes, streamlines, isolines or isosurfaces of scalar variables). In contrast to the standard stand-alone visualization packages for displaying the CFD data, the Google Earth provides a photo-realistic description (that includes numerous geographic information system (GIS) features such as the 3D terrain, buildings, vegetation, roads, water surfaces, soil, etc.) of the simulated domain, making the interpretation of the visualized datasets relatively easy and intuitive. Besides, the Google Earth provides an easy interface to record animations with fly-through effects of the selected datasets (an example of such animation is attached as the Supplementary File/Video). 

To demonstrate some capabilities of the newly developed CFD/CRD to Google Earth dataset conversion and visualization algorithms, we analyzed the flow and turbulent dispersion of reactive scalars (photochemical smog generation) for a neighborhood in the city of Rotterdam, The Netherlands. Here, the surface pollutant sources and incoming wind conditions were specified to match realistic traffic emissions and typical meteorological situations. Maps of the local velocity field at different heights were generated. On top of that, isolines and isosurfaces of the reactive scalars were shown. Furthermore, the discrete volume objects were used as a kind of the virtual probes/monitors/sensors making it possible to quantify the local pollution thresholds in a straightforward and efficient way. Finally, the fly-by animations (attached as supplementary material video) along the streets under study provided insights in depth into the local levels of pollution. Furthermore, apart from the 3D buildings natively provided by the Google Earth, the manually created geometry models of the simulation domain can also be imported (as shown in Figure 5). It means that the presented tool can also be used in the areas where the 3D obstacles (or buildings) utilized in the model do not exist in the Google Earth. 

Note that due to simplicity of the requested structure of the input data (spatial coordinates of the control volume nodes and values of vector and scalar components at the control volume centers) here presented tool can also be used for any other visualizations of the CFD/CRD model of environmental flow and pollution dispersion in a city-scale or neighborhood scale. Finally, we conclude that the presented approach of CFD/CRD conversion and visualization in the Google Earth proved to be a numerically efficient and user-friendly way of displaying results.

## Supplementary Materials

The following is available online at www.mdpi.com/1660-4601/14/3/247/s1, Video S1: “A tour along the Bergselaan street”.
